# iFish: predicting the pathogenicity of human nonsynonymous variants using gene-specific/family-specific attributes and classifiers

**DOI:** 10.1038/srep31321

**Published:** 2016-08-16

**Authors:** Meng Wang, Liping Wei

**Affiliations:** 1 Center for Bioinformatics, State Key Laboratory of Protein and Plant Gene Research, School of Life Sciences, Peking University, Beijing, P.R. China

## Abstract

Accurate prediction of the pathogenicity of genomic variants, especially nonsynonymous single nucleotide variants (nsSNVs), is essential in biomedical research and clinical genetics. Most current prediction methods build a generic classifier for all genes. However, different genes and gene families have different features. We investigated whether gene-specific and family-specific customized classifiers could improve prediction accuracy. Customized gene-specific and family-specific attributes were selected with AIC, BIC, and LASSO, and Support Vector Machine classifiers were generated for 254 genes and 152 gene families, covering a total of 5,985 genes. Our results showed that the customized attributes reflected key features of the genes and gene families, and the customized classifiers achieved higher prediction accuracy than the generic classifier. The customized classifiers and the generic classifier for other genes and families were integrated into a new tool named iFish (integrated Functional inference of SNVs in human, http://ifish.cbi.pku.edu.cn). iFish outperformed other methods on benchmark datasets as well as on prioritization of candidate causal variants from whole exome sequencing. iFish provides a user-friendly web-based interface and supports other functionalities such as integration of genetic evidence. iFish would facilitate high-throughput evaluation and prioritization of nsSNVs in human genetics research.

The rapid development of next-generation sequencing (NGS) technology and its wide application in disease gene discovery and clinical genetics brought both opportunities and challenges. A major challenge is how to assess the disease relevance of genomic variations on a large scale^1^. Single Nucleotide Variants (SNVs) are the most abundant type of variants in the human genome. On average each individual has over three million SNVs^2^. Although nonsynonymous SNVs (nsSNVs) account for more than half of mutations known to lead to genetic disorders^3^, unlike truncated mutations or frameshift mutations which usually tend to be damaging, the vast majority of the nsSNVs in the human genome are benign and do not lead to disease phenotypes^4^. Thus, accurately distinguishing pathogenic nsSNVs from neutral ones is critical in biomedical research and clinical genetics^5^,^6^. Because the sequenced variants are far too many for experimental studies, *in silico* methods to predict pathogenicity are often used to prioritize the variants for further experiments^1^,^4^.

The state-of-the-art *in silico* methods discriminate pathogenic nsSNVs from neutral ones by measuring sequence evolutionary conservation such as SIFT^7^, MAPP^8^, or by incorporating both sequence, structure and function features such as SAPRED^9^, MutPred^10^, SNPs&GO^11^, MutationTaster2^12^ and PolyPhen2^13^. These tools build and train a generic machine-learning classifier based on known pathogenic and neutral nsSNVs in the human genome. However, each gene has its own features, and genes in a gene family share similar features. Finding and using gene-specific/family specific attributes, rather than applying the same fixed attributes to assess all of the nsSNVs in the human genome, would be more biologically meaningful and help to improve the prediction performance.

In fact, gene-specific classifiers have been built for a small number of genes such as the causal genes for CHARGE syndrome (*CHD7*)^14^ and alternating hemiplegia of childhood (*ATP1A3*)^15^. Group-specific classifiers have been built for a group of six functionally related genes associated with hypertrophic cardiomyopathy (*MYH7*, *TNNT2*, *TPM1*, *TNNI3*, *MYPC3* and *MYL2*)^16^. They were found to achieve higher accuracy than generic classifiers^14^,^16^. However, it remains untested whether these are special cases or reflect a general rule. Gene-specific/family-specific classifiers have not been implemented or assessed at the whole genome scale. One possible reason was that in previous years there were not enough known pathogenic and neutral variants to train and test gene-specific/family-specific classifiers. Fortunately, largely driven by the high-throughput genotyping and sequencing technologies in the past decade, there are now a wealth of SNV data from large genetic studies and the 1000 Genomes Project^2^,^17^. Large number of known pathogenic SNVs are now stored in databases such as the Online Mendelian Inheritance in Men (OMIM)^18^, Human Gene Mutation Database (HGMD)^3^ and locus specific databases^19^. The abundant neutral and pathogenic SNV data makes it possible to build gene-specific/family-specific attributes and classifiers and test whether they can achieve better accuracies.

Here we reported our construction and rigorous evaluation of gene-specific and family-specific attributes and classifiers for the prediction of the pathogenicity of nsSNVs. We implemented the classifiers into a new online web server, iFish (integrated Functional inference of SNVs in human, http://ifish.cbi.pku.edu.cn/), which had flexible functionalities and user-friendly interfaces. We evaluated iFish on multiple independent benchmarking datasets and compared its performance with seven other state-of-the-art tools. We also implemented other user-friendly functionalities in iFish such as integration of users’ own collected data on certain genes to build their own gene specific classifiers.

## Results

### Overview of iFish

**Figure 1:**
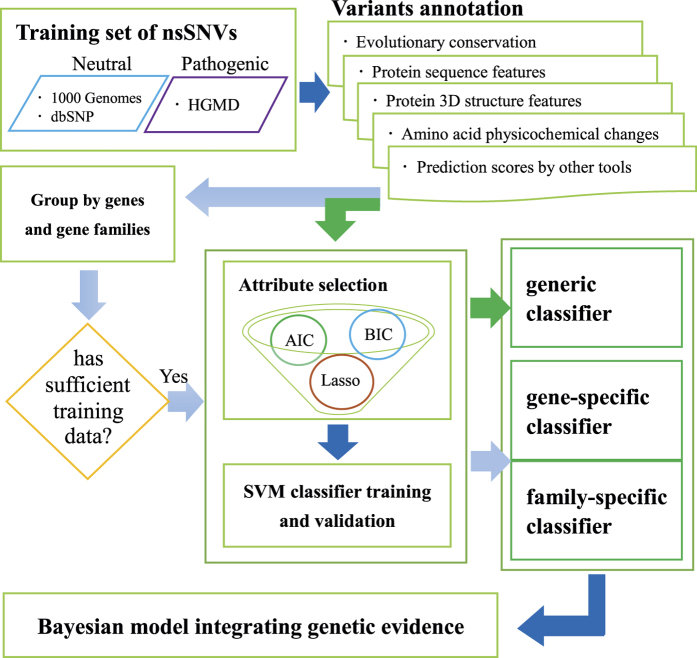
Overall framework of iFish. The arrows indicate the order of the five steps: training data collection, variants annotation, attribute selection, classifier construction, and genetic evidence integration. The light blue arrows indicate the steps for gene-specific/family-specific customized attribute selection and classifier construction. The green arrows indicate the steps for generic attribute selection and classifier construction. The dark blue arrows indicate shared steps.

iFish predicts the pathogenicity of nonsynonymous SNVs using gene and gene family specific attributes and classifiers (Fig. 1). 58,794 unique pathogenic nsSNVs on 2,877 disease related genes and 70,189 neutral nsSNPs on 15,552 genes were collected as training set. Each variant was annotated with 40 attributes that had been previously demonstrated informative for classification by our^9^ and other groups^13^ (Supp. Table S1). Several studies had demonstrated that the integration of prediction results from multiple tools resulted in a higher classification accuracy^20^,^21^. Therefore, prediction scores from widely used tools including SIFT, PolyPhen2 and MutationAssessor^22^ were included in the list of candidate attributes. The 40 attributes annotated for each variant can be grouped into five categories: evolutionary conservation, protein sequence features, protein structural features, amino acid physicochemical changes and prediction scores from other tools. Supp. Figure S1 showed the z-score distribution of all the collected pathogenic and neutral nsSNVs for each attribute. Unlike existing tools that make use of the same set of attributes to classify variants in all genes, different predictive attributes were automatically selected with AIC, BIC and Lasso method for different genes and gene families that have adequate pathogenic and neutral training nsSNVs (detailed in Methods). A linear support vector machine (SVM) classifier was constructed and trained using selected attributes for each customized gene and gene family to assert novel nsSNVs.

**Figure 2:**
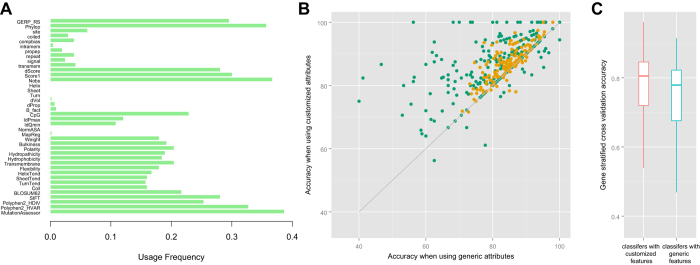
Evaluation of gene-specific and family-specific customized classifiers and comparison with generic classifiers. (**A**) The usage frequency of each candidate attributes in these customized classifiers. The y-axis represents the 40 candidate attributes, and the x-axis represents their usage frequency in all classifiers. (**B**) Comparison of cross-validation accuracies using the customized attributes (Y-axis) *vs.* using the generic attributes (X-axis). Each point represents a gene (green) or a gene family (orange) that had enough training SNVs for customized attributes and classifiers. (**C**) Evaluation of the gene-family specific classifiers using gene stratified cross validation (leave variants on one gene out). The gene-family specific classifiers with customized features achieved higher accuracies than the classifiers using generic features.

Finally, 254 genes had sufficient training nsSNVs for gene-specific customized attribute selection. Another 152 gene families, covering additional 5,731 genes, had sufficient training nsSNVs for family-specific customized attribute selection. We also built a generic classifier to predict the effect of variants in genes that did not have sufficient training variants to build customized classifiers. The generic attributes were selected based on half of all training nsSNVs (see Methods). The list of selected attributes for genes and gene families were available on the iFish website and Supplementary Data S1. A total of 28 attributes were selected as generic attributes. In contrast, most genes and families had a relatively small number of customized attributes selected and over 44.9% of genes and families had only 1–4 customized attributes selected (Supp. Figure S2). The usage frequency of the different attributes were shown in Fig. 2A.

As an example of gene specific attributes selection in iFish, for the *DOK7* gene which is associated with congenital myasthenic syndrome, four attributes including GERP conservation score, hydrophobicity, transmembrane tendency and turn tendency of amino acids were selected. This gene encodes a pleckstrin homology domain for membrane association and a phosphotyrosine binding domain and its protein sequence is conserved among many species^23^. It had been shown that mutations on *DOK7* gene can affect salt bridge formations, hydrophobic space and backbone torsion angles^24^. The four customized attributes covered these functional effects and were informative for variants pathogenicity prediction.

Together with functional effect predictions, iFish supports the integration of genetic evidences such as co-segregation with disease in a family and case-control associations. These evidences are quantified and integrated in iFish with a Bayesian model (see methods), which yields a unified posterior probability that the SNV is pathogenic given all of the available evidences. Incorporating genetic evidences were previously shown to be effective in discriminate pathogenic variants from neutral ones in cancer genes such as *BRCA1* and *BRCA2* 25,26.

### Evaluation and comparison of gene-specific, family-specific and generic classifiers

The performance of all the SVM classifiers were first evaluated by ten-fold cross validation. The generic classifier achieved ten-fold cross-validation accuracy of 0.83 on the training set. In comparison, gene-specific and family-specific customized classifiers achieve significantly higher accuracies than the generic classifier on nsSNVs in most corresponding genes and families (Fig. 2B, p-value < 10^−15^, Wilcoxon Signed-rank Test), with a mean accuracy of 0.87. Specifically, 71.43% (290) of the customized classifiers achieved higher accuracy than the generic classifier on nsSNVs in the corresponding genes and families and 20.44% (83) of the customized classifiers achieved the same accuracy as the generic classifier. Only 8.13% (33) of the customized classifiers achieved lower accuracy than the classifier using generic features, and most of the differences were small as illustrated by the points below the diagonal in Fig. 2B. We also performed gene-stratified cross-validation (by leaving out all variants on one gene at a time) for the family-customized classifiers, and found that they achieved significantly higher accuracies than that using generic features (Fig. 2C, p-value < 10^−13^, Wilcoxon Signed-rank Test). We then evaluated and compared the performance of the classifiers on nsSNVs in the corresponding genes and gene families in the benchmark datasets NovelVar. Customized classifier method achieved the classification accuracy of 0.74, an increase of 0.01 over the generic classifier. These results demonstrated that customized classifiers built with gene-specific and family-specific attributes achieved high prediction accuracy and outperformed the generic classifier.

### Performance evaluation of iFish and comparison with other tools

We next evaluated the performance of iFish, applying the customized classifiers whenever possible and the generic classifier on all other nsSNVs. We compared iFish with seven other widely employed tools, SIFT, PolyPhen2, MutationAssessor, CADD^27^, MutationTaster2, FATHMM^28^ and Condel^20^. We employed three stringent independent test datasets (described in Methods and Table 1). To evaluate the actual ability of the prediction tools in discriminating novel pathogenic variants from neutral ones, the benchmark test sets should be carefully selected to avoid two types of circularity^29^. Type 1 circularity is the overlap between training variants and testing variants. We constructed an independent test dataset, named NovelVar, to avoid the type 1 circularity. Type 2 circularity occurs when all variants in the same gene are jointly annotated as pathogenic or neutral, which may mislead tools that rely on gene-level information to overfit^29^. To test whether these tools were confounded by type 2 circularity, we constructed two independent test sets, SwissvarFilteredMix and VaribenchSelectedPure, based on public benchmark test datasets SwissVar and Varibench^30^. The SwissvarFilteredMix test set contained variants in “mix” genes (genes with both pathogenic and neutral variants) and the VaribenchSelectedPure set was composed of variants on “pure” genes (genes with all variants labeled as pathogenic or all labeled as neutral). If a prediction tool was affected by type 2 circularity, then its accuracy would be spuriously inflated on the VaribenchSelectedPure test dataset compared to that on the SwissvarFilteredMix test dataset^29^.

**Figure 3:**
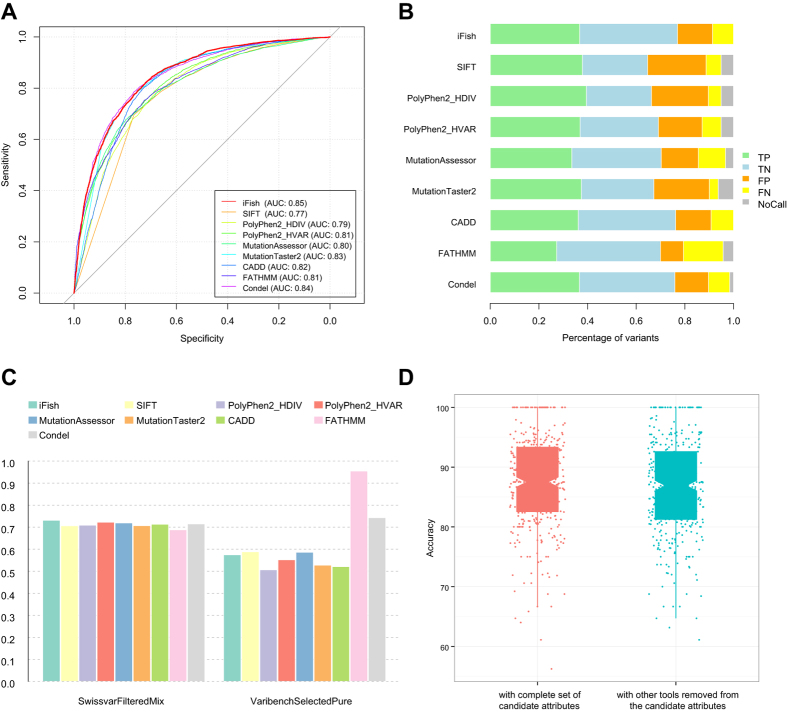
Performance evaluation of iFish and comparison with other tools. (**A**) ROC curves on NovelVar test dataset of iFish, SIFT, PolyPhen2 trained by HumDiv and HumVar, MutationAssessor, MutationTaster2, CADD, FATHMM and Condel. (**B**) Comparison of true positives (TP), true negatives (TN), false positives (FP), false negatives (FN), and not called (NoCall) variants of these tools on NovelVar test dataset. (**C**) AUCs of these tools on test sets composed by only variants on “mix” genes (SwissvarFilteredMix) and that only had variants on “pure” genes (VaribenchSelectedPure). iFish archived the best AUC on SwissvarFilteredMix test set. And unlike FATHMM, the AUC of iFish and other tools did not spuriously inflated on VaribenchSelectedPure. (**D**) Boxplot of the cross validation accuracies of each customized classifier in iFish and compared with the classifiers when prediction results of SIFT, MutationAssessor, PolyPhen2 trained by HumDiv and HumVar were removed from the candidate attributes set. These tools contributed to the improvement of the accuracies. However, when these tools were excluded, most classifiers still remained high accuracy.

ROC analysis showed that iFish achieved the highest AUC (0.85) on NovelVar (Fig. 3A). iFish also achieved the highest prediction accuracy of 76.99% and the best MCC of 0.54 on NovelVar, with both high sensitivity and specificity (Fig. 3B and Table 2). By default, iFish utilized customized prediction cutoff for each classifier that maximize the sum of sensitivity and specificity. This cutoff can be reset by the user in the iFish web tool to achieve different desired sensitivities and specificities for different research questions. With the default configuration, although the sensitivity of iFish was a little lower than SIFT, PolyPhen2 and MutationTaster2, its specificity was much higher than that of the other tools (Table 2). If only the generic classifier is used, the AUC of iFish was 0.84 on NovelVar which was the same as Condel and better than the other tools. Details about the prediction results of the tools and the NovelVar dataset were provided in Supp. Data S2.

iFish also archived the highest AUC of 0.73 on the independent test set SwissvarFilteredMix, followed by PolyPhen2 trained by HumVar (AUC: 0.72), MutationAssessor (AUC: 0.72), Condel (AUC: 0.71), CADD (AUC: 0.71), PolyPhen2 trained by HumDiv (AUC: 0.71), SIFT (AUC: 0.71), MutationTaster2 (AUC: 0.70) and FATHMM (AUC: 0.69), shown in Fig. 3C. Furthermore, the performance of iFish was not inflated when assessing variants on “pure” genes in VaribenchSelectedPure (Fig. 3C). It is useful to note that the performance of the tools on VaribenchSelectedPure cannot be compared directly because this dataset was biased and the labels of variants in this dataset were at least partially artificial^29^. It was only constructed for testing whether a prediction method was confounded by type 2 circularity. Details about the prediction results of all the tools and the SwissvarFilteredMix and VaribenchSelectedPure test datasets were provided in Supp. Data S3 and Supp. Data S4, respectively.

Taken together, the prediction results on the three independent test datasets demonstrated that the accuracy of iFish was high and not confounded by type 1 or type 2 circularity. Compared to iFish, MutationTaster2 and Condel had similar AUC on NovelVar. But the performance of MutationTaster2 was slightly worse on SwissvarFilteredMix (Fig. 3C) and did not show consistent high performance over the other tools. This indicated that the high performance of MutationTaster2 on NovelVar might be confounded by type 1 circularity to certain extent. MutationTaster2 used private training variants which were not publicly available and may overlap with NovelVar. The performance of Condel got inflated a little when predicting variants on ‘pure’ genes than that on ‘mix’ genes, indicating that its high performance may be due to type 2 circularity^29^ to certain extent. Another tool, FATHMM, performed the worst on SwissvarFilteredMix but got a spuriously highest AUC of 0.95 on VaribenchSelectedPure (Fig. 3C), suggesting that FATHMM was confounded by type 2 circularity.

Since iFish incorporated the prediction scores from existing tools (SIFT, MutationAssessor, and PolyPhen2 trained by HumDiv and HumVar) into the classifiers, we next evaluated the effect of incorporating these tools on the iFish performance, by removing scores of these tools from the list of candidate attributes and re-running attribute selection and classifier construction. Even after these tools were removed from the candidate attribute set, iFish still achieved a high AUC of 0.82 in ROC analysis on NovelVar (Supp. Data S2). For 35.47% of the customized classifiers, the cross validation accuracies remained unchanged whether or not the other tools were included in the candidate attribute set (Fig. 3D). These results demonstrated that candidate attributes other than these prediction tools contributed substantially to the high accuracy of iFish, whereas inclusion of these tools further improved the accuracy (Fig. 3D. p-value = 0.005, Wilcoxon Signed-rank Test). Thus the final version of iFish incorporated these tools into the classifier.

### Application to discover disease causal gene from next-generation whole exome sequencing data

We demonstrated the utility of iFish in the discovery of disease causal genes and mutations from two whole exome sequencing datasets. The candidate nsSNVs in four individuals affected by Miller Syndrome (MIM: 263750) were ranked by their probability of being pathogenic by iFish and five other tools. We did not run FATHMM here because it was significantly confounded by type 2 circularity. Two mutations in *DHODH* which were the correct causal mutations in the correct gene^31^ ranked among top 3 by iFish with scores of 0.98 and 0.97, respectively (Supp. Table S2). The single validated neutral nsSNV in this gene (rs3213422:A > C)^32^ was predicted to be neutral by iFish with a score of 0.53. In contrast, the result from SIFT ranked 199 mutations in 157 genes (including *DHODH*) as equally most pathogenic. MutationAssessor had reasonable performance and ranked the two causal mutations in *DHODH* among its top 10 list (Supp. Table S3). PolyPhen2 ranked 117 mutations in 102 genes (including *DHODH*) as equally most pathogenic when trained by HumDiv and 30 mutations in 27 genes (including *DHODH*) as equally most pathogenic when trained by HumVar. CADD did not include any mutation in *DHODH* in its top 10 list. MutationTaster2 ranked 62 mutations in 56 genes as equally most pathogenic, not including *DHODH*. Condel ranked only one causal mutation in its top 10 list. Thus, iFish had the best performance in discovering the causal gene and mutations.

We then applied iFish and the other tools to prioritize candidate nsSNVs from the whole exome sequencing data of the Alternating Hemiplegia of Childhood (AHC) families, whose causal gene was discovered to be *ATP1A3* 15,33,34. One causal mutation in *ATP1A3* ranked the first by iFish, and both of the other two causal mutations in this gene ranked 12th by iFish (Supp. Table S4). SIFT ranked 135 mutations in 104 genes (including *ATP1A3*) as equally most pathogenic. MutationAssessor ranked one mutation in *ATP1A3* among its top 10 list (Supp. Table S5). PolyPhen2 trained by HumDiv ranked 78 mutations in 69 genes (including *ATP1A3*) as equally most pathogenic, and PolyPhen2 trained by HumVar ranked 19 mutations in 16 genes (including *ATP1A3*) as equally most pathogenic. CADD and Condel included no mutation in *ATP1A3* in their top 10 lists. MutationTaster2 ranked 38 mutations in 29 genes (including *ATP1A3*) as equally most pathogenic. Thus, compared with the other tools, iFish showed the strongest signal indicating the causal mutations and causal gene from next-generation sequencing data.

## Discussion

Here we presented a new tool, iFish, that could predict the pathogenicity of human nsSNVs with increased accuracy. We demonstrated that iFish outperformed other existing tools on stringent independent benchmarking datasets. iFish has an user-friendly and flexible web-based interface that allows users to analyze large sets of nsSNVs rapidly. It supports the integration of genetic evidence into the prediction of pathogenicity using a Bayesian model. It also facilitates registered users to construct their own gene-specific classifiers using their private variants, which is not available in other existing tools.

Grimm *et al*. cautioned that two types of circularity may spuriously increase the prediction accuracies in the evaluation of pathogenicity prediction tools^29^. Here we constructed independent benchmark test datasets with no overlap with the training datasets to avoid type 1 circularity, and showed that iFish achieved high accuracies and outperformed other existing tools. Type 2 circularity was often ignored in previous evaluation of prediction tools^29^. Here we constructed one test dataset on “mixed” genes and another on “pure” genes, and showed that the high accuracy of iFish was not confounded by type 2 circularity.

The diverse characteristics of different genes and gene families were largely overlooked by previous predicting systems which built a single genome-wide classifier and employed a single genome-wide cutoff. Recently, Itan *et al*. proposed a method with gene-customized cutoff and demonstrated that the gene-level prediction system outperformed that using uniform cutoff^35^. However, this method employed the unified scoring system for all the genes and did not take gene-specific features into consideration. We built gene/family-specific classifiers that not only had gene/family-specific prediction cutoff but also utilized gene/family-specific features. We demonstrated that most gene-specific and family-specific customized classifiers achieved higher prediction accuracies than the generic classifier. The increase in prediction accuracy was in part due to the customized attributes selected for each gene and family. Proper attribute selection is critical for improving the performance of machine learning based methods. Each gene has its own characteristics. Not all generic attributes were informative for classification of variants in every gene. Selecting the most predictive customized combination of attributes captured the key features of different genes and gene families. In fact, Gene Ontology (GO) enrichment analysis showed that genes with a certain attribute selected in iFish were enriched in GO terms that were related to that attribute, whereas genes without that attribute selected were not enriched in these GO terms (Supp. Table S6). For instance, genes with the ‘Intramembrane’ attribute selected were enriched in GO terms ‘integral component of membrane’, ‘intrinsic component of membrane’ and ‘membrane part’. Genes with the ‘site’ attribute selected were enriched in GO terms ‘protein binding’, ‘ligase activity’ and ‘receptor binding’. These results suggested that the customized attributes selected for genes reflected gene-specific features.

A limitation of building gene-specific and family-specific customized models is that the number of customized models was limited by the amount of available training pathogenic and neutral variants in a gene and gene family. Although tens of thousands nsSNVs with known relevance to diseases were collected, they mainly concentrated on a small subset of genes and gene families. Most of genes and gene families had too few training SNVs to build customized classifiers. Customized models have higher risks of overfitting, and thus more careful and extensive evaluations, as we had conducted here, are required. Another challenge, faced by not only iFish but also other prediction models, is that some SNVs in the training set might be mislabeled. Variants labeled as disease mutation in HGMD database might not be the real causal mutation. Instead they might be in linkage disequilibrium with the underlying causal mutation. The common variants in individuals without disease phenotype are assumed to be neutral. However, these variants may also give rise to functional alterations or late-onset diseases. These could reduce the accuracy of the classifiers, especially the customized classifiers which have a smaller training set. This situation would gradually improve as more data and knowledge become available on the functional effects of genetic variants.

The advances of human genetic research, fueled by next-generation sequencing technologies, will continue to increase the number of known pathogenic and neutral nsSNVs. We anticipate more customized classifiers to be built for increasing number of genes and gene families. As a result, the prediction accuracy of iFish will continue to increase. We will continue to update iFish to make it a useful tool for human genetic and genomic studies.

## Methods

### Collection of neutral and pathogenic nsSNVs as training dataset

Variants from the Phase 3 of the 1000 Genomes project^36^ were downloaded from the ftp site (ftp://ftp.1000genomes.ebi.ac.uk/) in VCF format. The raw variants were filtered such that only common SNVs with a population allele frequency of at least 1% were retained. In addition, common SNVs with a population allele frequency of at least 1% in dbSNP138 were retrieved from the UCSC genome browser (http://genome.ucsc.edu/). These two sets of common variants were combined with duplicated items removed and used as the “neutral” training SNVs. The pathogenic SNVs were obtained from HGMD (release 2014.4) database with the tag ‘DM’. Some pathogenic mutations in HGMD were also present in the common variants in the 1000 Genomes Project or dbSNP. These variants were removed from the training SNVs. The collected SNVs were then annotated by ANNOVAR^37^ and mapped to the genes (UCSC KnownGene) in which they were located. Only nonsynonymous SNVs were retained. The genomic coordinates of the SNVs were based on human reference genome GRCh37.

### Calculation of the Sequence and Structural Attributes for the Variants

GERP^38^ scores for each variant were obtained from the precompiled results on the website (http://mendel.stanford.edu/SidowLab/downloads/gerp/). Phylop^39^ conservation scores were downloaded from the UCSC genome browser (https://genome.ucsc.edu/). The protein sequence features and protein 3D structure features were annotated using PolyPhen2 (version 2.2.2). Physicochemical changes of amino acids were based on values given by ProtScale (http://web.expasy.org/protscale/). The missing values in each feature were replaced by the mode value of that feature. The raw values for each attribute were scaled to obtain z-scores, which were used in subsequent attribute selection, model training and validation.

### Variants grouping by genes and gene families

To build customized classifiers for genes and gene families when possible, all of the nsSNVs in the training dataset were grouped based on the genes and gene families in which they were located. The list of gene families and the genes they include were based on the HGNC Gene Families/Groupings Nomenclature (http://www.genenames.org/genefamily.html). In order to build a customized classifier, we required that (1) a gene should have at least 15 training nsSNVs and a gene family should have at least 30 training nsSNVs. This criteria resulted in 794 genes containing 41,117 pathogenic nsSNVs and 5,635 neutral nsSNVs and 261 gene families containing 39,955 pathogenic nsSNVs and 36,219 neutral nsSNVs. (2) The ratio of pathogenic to all variants should be between 0.2 and 0.8. Adding this criteria resulted in a final set of 254 genes containing 4,073 pathogenic nsSNVs and 2,548 neutral nsSNVs and 152 gene families containing 21,458 pathogenic nsSNVs and 23,884 neutral nsSNVs.

### Attribute selection and classifiers construction

Attribute selection is an important step to reduce the risk of overfitting. Three methods were employed to select attributes from the attribute list: logistic regression with Akaike Information Criterion (AIC) model selection, logistic regression with Bayesian Information Criterion (BIC) model selection, and least absolute shrinkage and selection operator (LASSO) regression. For each gene/family that had enough balanced training variants, attributes were selected with AIC, BIC, and LASSO, respectively, and for each set of selected attributes, a support vector machine (SVM) classifier was trained with linear kernel function, implemented using libSVM^40^, and validated by ten-fold cross validation. The SVM classifier that had the best cross-validation accuracy was used as the final classifier for that gene/family and the corresponding attributes set was designated as the final attributes set for that gene/family. To build the generic classifier, all the training nsSNVs were randomly split into two parts with equal number of variants. One part was used to select genome-wide generic attributes and the other part was used to train the genome-wide generic SVM classifier. The parameters in all the classifiers were chosen using grid search to maximize the cross validation accuracy of classification. The customized classifiers and the generic classifier were combined into iFish.

### Performance evaluation and comparison with other tools

The accuracies of the customized classifiers and generic classifier were first evaluated using ten-fold cross validation on the training set. For the family-specific classifiers, we also performed gene-stratified cross validation on the training set by leaving out all variants on one gene at each time. For 30 gene families that had one single gene containing more than 50% total variants of this family, the gene stratified cross validation was not performed since the distribution of the variants was too unbalanced. Next we compared the performance of iFish *vs.* other state-of-the-art tools (SIFT, PolyPhen2 trained by HumDiv and HumVar, respectively, MutationAssessor, CADD, MutationTaster2, FATHMM and Condel) on three independent test sets. Accuracy was measured using Receiver Operating Characteristic (ROC) analysis^41^. The classification accuracy, false discovery rate (FDR), sensitivity, specificity, and Matthews Correlation Coefficient (MCC) were also evaluated and compared. For iFish and CADD, the predicted class for nsSNVs was obtained by maximizing the sum of the sensitivity and specificity. For SIFT and MutationAssessor, the classification cutoff was set to 0.05 and 1.9, respectively, as recommended^22^,^42^. For Polyphen2, MutationTaster2, FATHMM and Condel, the predicted classes were included in their outputs.

We constructed three stringent test datasets. NovelVar was comprised of pathogenic variants in HGMD 2015.3 release and common variants (AF ≥ 1%) in dbSNP142 that did not overlap with iFish training variants nor the training variants of PolyPhen2 (HumDiv and HumVar) which was used as a candidate attribute in iFish. Scores from SIFT and MutationAssessor were also used as candidate attributes in our tool, but they were based on measuring the evolutionary conservation and were not trained using known pathogenic and neutral variants^7^,^43^. We constructed the SwissVarFilteredMix test dataset by (1) removing from SwissVar (release 2015.9, http://www.uniprot.org/docs/humsavar) all variants overlapping with iFish training dataset or PolyPhen2 training dataset, and (2) selecting only variants in SwissVar located in “mix” genes. We constructed the VaribenchSelectPure dataset by (1) removing from VaribenchSelected all variants overlapping with iFish training dataset or PolyPhen2 training dataset, and (2) selecting only variants in VaribenchSelected located in “pure” genes.

We compared the performance of iFish and the other tools on the prioritization of disease-causing candidate mutations from two next-generation sequencing datasets. The first was a commonly used benchmark dataset from the whole exome sequencing of four unrelated individuals affected by Miller Syndrome (MIM: 263750), obtained from NCBI dbGaP (http://www.ncbi.nlm.nih.gov/gap) with accession number phs000244.v1.p1. Miller Syndrome was known to be caused by mutations in *DHODH*^31^. The second was the whole exome sequencing data of six unrelated children affected by Alternating Hemiplegia of Childhood (AHC, MIM: 614820) together with both unaffected parents of three of these affected children (dbGaP Study Accession: phs000660.v1.p1)^15^. AHC was a rare dominant disorder caused predominantly by *de novo* mutations in *ATP1A3.* For both datasets, the raw reads were trimmed to remove sequencing adaptors; reads of low quality were filtered; and the remaining reads were mapped to GRCh37 reference genome using BWA^44^. Variants were called by GATK^45^ following the recommended best practices^46^. For the Miller Syndrome dataset, observed nsSNVs on genes on which all four affected individuals carried nsSNVs were included as candidate variants, resulting in a total of 2,365 nsSNVs in 1,275 genes. For the AHC dataset, nsSNVs on genes on which at least two affected individuals carried nsSNVs and that were absent in the unaffected parents were included as candidate variants, resulting in a total of 710 nsSNVs in 400 genes. The causal mutations of Miller Syndrome and AHC were removed from iFish training set.

### Bayesian model to integrate genetic evidence

Genetic evidences (GE) were integrated with the prediction of pathogenicity by SVM into a naïve Bayesian model. Each nsSNV was considered as either pathogenic (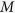) or neutral (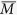). The probability of pathogenicity (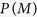) predicted by the SVM classifier was taken as the prior probability of pathogenicity for the nsSNV. 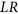
was the likelihood ratio of observing the genetic evidence under the assumption that the nsSNV was pathogenic versus under the assumption that the nsSNV was neutral. We calculated the posterior probability that a mutation was pathogenic given all of the available genetic evidences as


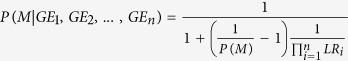


Co-segregation was quantified by calculating the likelihood ratio as described by Thompson *et al*.^47^. Likelihood ratios of case-control association were derived by employing Bayesian statistical methods for association studies, as described by Stephens and Balding^48^.

### Implementation of iFish web-based application

Gene-specific, family-specific, and the generic classifiers were integrated into iFish with a web-based user interface, freely available at http://ifish.cbi.pku.edu.cn. iFish takes one or more nsSNVs as input, specified by the chromosome, position, reference allele and alternative allele of each nsSNV. The variant calling results from next-generation sequencing in the standard Variant Call Format (VCF) could be directly used as input. Genetic evidence, when available, could be provided in VCF, together with the pedigree file in family-based co-segregation analysis or the phenotype of each individual in case-control association studies.

The annotation and prediction results given by iFish could be retrieved and downloaded in the web browser. To enable iFish making rapid predictions, we had enumerated all possible nsSNVs in the human genome, precompiled all annotations, and stored them in a local Hash database. The customized attributes selected for each gene and gene family could be browsed in the web browser. For users who have significant amount of private nsSNV training data on genes of their interest, iFish supports the users to build their own customized classifiers using their private training data.

## Additional Information

**How to cite this article**: Wang, M. and Wei, L. iFish: predicting the pathogenicity of human nonsynonymous variants using gene-specific/family-specific attributes and classifiers. *Sci. Rep.* **6**, 31321; doi: 10.1038/srep31321 (2016).

## Supplementary Material

Supplementary Information

Supplementary Dataset 1

Supplementary Dataset 2

Supplementary Dataset 3

Supplementary Dataset 4

## Figures and Tables

**Table 1:** Summary of training and testing nsSNVs datasets.

	Name	Source	Number of pathogenic nsSNVs	Number of neutral nsSNVs	Removed variants overlapping with	Notes
**Training variants**	iFishTrainVar	Pathogenic: HGMD 2014.4	58,794	70,189	—	Common nsSNVs (AF 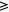 1%) in 1000 Genomes and dbSNP were used as neutral variants.
		Neutral: 1000 Genomes dbSNP138				
**Testing variants**	NovelVar	Pathogenic: HGMD 2015.3	4,044	4,868	iFishTrainVar	Common nsSNVs (AF 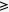 1%) in dbSNP were used as neutral variants.
		Neutral: dbSNP142			PolyPhen2 training variants	
	SwissvarFilteredMix	UniProt	1,217	1,324	iFishTrainVar	Only variants on “mix” genes were included.
					PolyPhen2 training variants	
	VaribenchSelectedPure	Varibench	2,144	3,777	iFishTrainVar	Only variants on “pure” genes were included.
					PolyPhen2 training variants	

**Table 2:** Performance evaluation and comparison using independent test set (NovelVar) of iFish, SIFT, MutationAssessor, PolyPhen2 trained by HumDiv and HumVar, CADD, MutationTaster2, FATHMM and Condel.

	Accuracy(%)	FDR(%)	Sensitivity(%)	Specificity(%)	MCC
iFish	76.99	28.16	81.06	73.60	0.54
SIFT	68.15	38.81	86.00	52.66	0.41
PolyPhen2 HDIV	69.81	37.18	88.22	53.37	0.44
PolyPhen2 HVAR	72.84	32.71	82.56	64.15	0.47
Mutation Assessor	72.70	31.23	75.03	70.69	0.46
Mutation Taster2	71.78	37.81	91.08	56.68	0.49
CADD	76.20	28.76	79.75	73.25	0.53
FATHMM	73.07	25.77	62.66	81.78	0.45
Condel	76.74	27.41	80.77	73.20	0.54

FDR: False Discovery Rate. MCC: Matthews Correlation Coefficient.
