# Supplementary material for: iFish: predicting the pathogenicity of human nonsynonymous variants using gene-specific/family-specific attributes and classifiers
Source: Sci Rep. 2016 Aug 16;6:31321. doi: 10.1038/srep31321 (PMC4985647; doi:10.1038/srep31321)
Supplement: Supplementary Information [file srep31321-s1.pdf]

Supplementary Figures and Tables for

iFish: predicting the pathogenicity of human nonsynonymous variants using gene-specific/family-specific attributes and classifiers

Meng Wang[1] and Liping Wei[1, *]

[1]Center for Bioinformatics, State Key Laboratory of Protein and Plant Gene Research, School of Life Sciences, Peking University, Beijing, P.R. China

[*]Correspondence to: Liping Wei, School of Life Sciences, Peking University, Beijing, 100871, P.R. China. E-mail: weilp@mail.cbi.pku.edu.cn

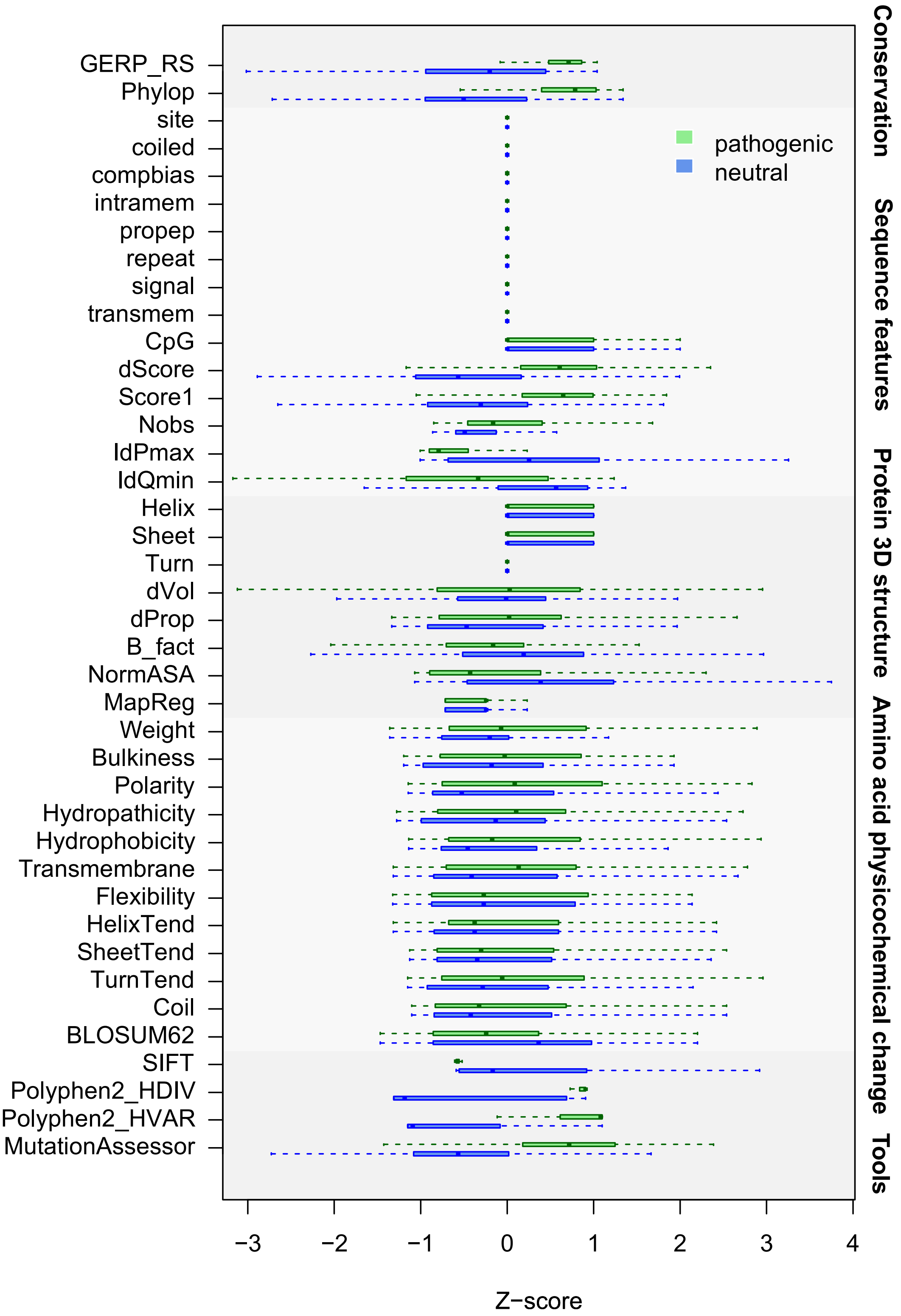

Supplementary Figure S1. Z-score distribution of all the pathogenic (green) and neutral (blue) nsSNVs in the training set for the 40 attributes in the candidate attributes set.

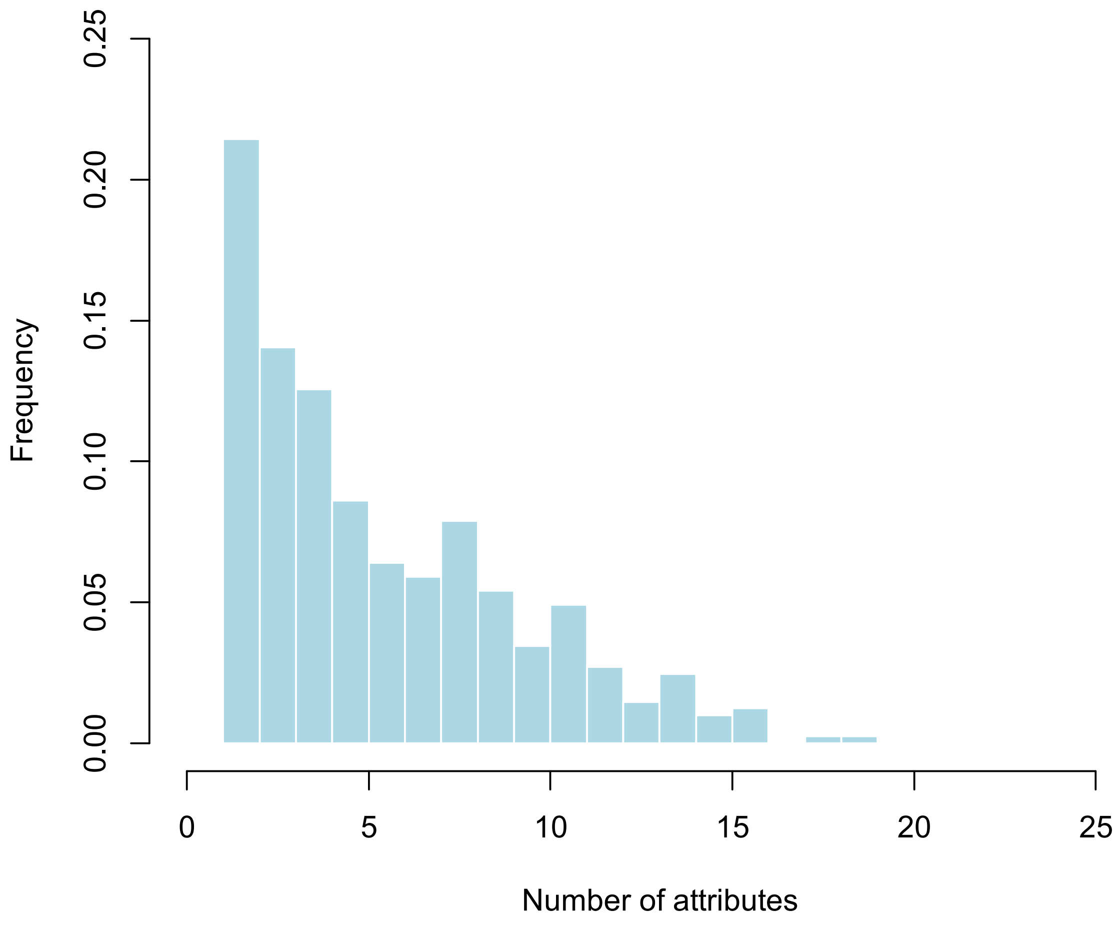

Supplementary Figure S2. Number of attributes remaining in each customized classifier after attribute selection.

Supplementary Table S1. Summary of the attributes annotated for each variant. GERP conservation scores were downloaded from its website (http://mendel.stanford.edu/SidowLab/downloads/gerp/). Phylop scores were downloaded from UCSC genome browser (https://genome.ucsc.edu/). Protein sequence features and 3D structure features were derived from PolyPhen2 (http://genetics.bwh.harvard.edu/pph2/) annotations. The value of amino acids physicochemical changes were based on ProtScale (http://web.expasy.org/protscale/).

	Attribute	Description
**Conservation scores**	GERP++_RS	GERP++ RS conservation score
	Phylop	Phylop conservation score
**sequence features**	site	SITE annotation in UniProt
	coiled	whether the substitution occurs in coiled region
	compbias	whether the substitution occurs in compositional bias region
	intramem	whether the substitution occurs in intramembrane region
	propep	whether the substitution occurs in propeptide region
	repeat	whether the substitution occurs in repeat region
	signal	whether the substitution occurs in signal peptide region
	transmem	whether the substitution occurs in transmembrane region
	CpG	whether substitution changes CpG context
	dScore	difference of PSIC scores of amino acid substitution
	Score1	PSIC score of wild type amino acid
	Nobs	number of amino acids observed at the substitution sites in a multiple sequence alignment
	IdPmax	maximum congruency of the mutant amino acid in a multiple sequence alignment
	IdQmin	sequence identity with the closest homologue
**Protein 3D structure features**	Helix	whether the substitution occur in helix secondary structure
	Sheet	whether the substitution occur in sheet secondary structure
	Turn	whether the substitution occur in turn secondary structure
	dVol	change in residue side chain volume

	dProp	change in surface solvent accessibility
	B-fact	normalized B-factor for the position
	NormASA	normalized accessible surface area
	MapReg	region of the phi-psi map
**Physicochemical change of amino acid**	Weight	change of residue molecular weight by the substitution
	Volume	change of residue volume by the substitution
	Polarity	change of residue polarity by the substitution
	Hydropathicity	change of residue hydropathicity by the substitution
	Hydrophobicity	change of residue hydrophobicity by the substitution
	TransmemTend	change of residue transmembrane tendency by the substitution
	Flexibility	change of residue flexibility by the substitution
	Helix tendency	change of residue helix tendency by the substitution
	Sheet tendency	change of residue sheet tendency by the substitution
	Turn tendency	change of residue turn tendency by the substitution
	Coil tendency	change of residue coil tendency by the substitution
	BLOSUM62	BLOSUM62 substitution score
**Score of other tools**	SIFT	http://sift.jcvi.org/
	MutationAssessor	http://mutationassessor.org/
	PolyPhen2_HDIV	http://genetics.bwh.harvard.edu/pph2/
	PolyPhen2_HVAR	http://genetics.bwh.harvard.edu/pph2/

Supplementary Table S2. Top mutations identified by iFish for the list of candidate variants from the Miller Syndrome whole exome sequencing data. The variants were ranked based on their probability of pathogenicity given by iFish. The genome position was based on GRCh37.

Rank	Chr	Variant	Gene	Prob
1	16	g.72048540C>T	DHODH	0.98
1	6	g.29523952A>G	UBD	0.98
3	16	g.72057435C>T	DHODH	0.97
3	17	g.8137826G>A	CTC1	0.97
5	12	g.48919659T>C	OR8S1	0.94
6	2	g.178565913T>C	PDE11A	0.93
6	11	g.5841926G>A	OR52N2	0.93
8	1	g.247614896A>C	OR2B11	0.92
8	12	g.21028208G>C	SLCO1B3	0.92
8	4	g.110605702C>A	CCDC109B	0.92

Supplementary Table S3. Top 10 mutations identified by MutationAssessor for the list of candidate variants from the Miller Syndrome exome. The variants were ranked based on the scores given by MutationAssessor. The genome position was based on GRCh37.

Rank	Chr	Variant	Gene	Prob
1	12	g.52966428G>C	KRT74	4.7750
2	16	g.72050942G>A	DHODH	4.6400
3	1	g.247614896A>C	OR2B11	4.5200
4	1	g.248844959T>C	OR14I1	4.4300
4	10	g.16961995A>C	CUBN	4.4300
6	11	g.7022160A>G	ZNF214	4.3900
7	16	g.72048540C>T	DHODH	4.2550
8	11	g.5841926G>A	OR52N2	4.0400
9	11	g.55563336A>T	OR5D14	4.0300
10	11	g.62294309C>T	AHNAK	4.0250

Supplementary Table S4. Top mutations identified by iFish for the list of candidate variants from the AHC whole exome sequencing data. The variants were ranked based on their probability of pathogenicity given by iFish. The genome position was based on GRCh37.

Rank	Chr	Variant	Gene	Prob
1	19	g.42472989C>A	ATP1A3	0.95
2	14	g.95088683T>C	SERPINA3	0.94
3	4	g.89052265C>T	ABCG2	0.93
3	7	g.146536932A>G	CNTNAP2	0.93
3	22	g.41574953T>A	EP300	0.93
6	17	g.72860371G>A	FDXR	0.91
7	11	g.1084362G>A	MUC2	0.90
7	3	g.40453442G>T	ENTPD3	0.90
7	4	g.6577044A>G	MAN2B2	0.90
7	7	g.48318149T>G	ABCA13	0.90
7	17	g.4675233T>C	TM4SF5	0.90
12	19	g.42474557C>T	ATP1A3	0.89
12	19	g.42490329G>T	ATP1A3	0.89

Supplementary Table S5. Top mutations identified by MutationAssessor for the list of candidate variants from the AHC exome sequencing. The variants were ranked based on the scores given by MutationAssessor. The genome position was based on GRCh37.

Rank	Chr	Variant	Gene	Prob
1	12	g.52981442C>A	KRT72	4.7600
2	2	g.179478967T>C	TTN	4.7400
3	10	g.16961995A>C	CUBN	4.4300
4	7	g.146536932A>G	CNTNAP2	4.2600
5	19	g.42472989C>A	ATP1A3	4.1000
6	11	g.55135964T>C	OR4A15	3.8800
6	8	g.134030167C>T	TG	3.8800
8	2	g.179479067C>T	TTN	3.8700
9	1	g.22176542G>A	HSPG2	3.7850
10	11	g.1084362G>A	MUC2	3.6100

Supplementary Table S6. The gene ontology (GO) enrichment results showed that the attributes selected in iFish for each gene and gene family reflected gene function features and are biologically meaningful. For each attribute that indicates biological functions, genes that had gene-specific or family-specific classifiers in which this attribute was utilized were tested to find the enriched GO terms, and evaluated whether these GO terms were relevant to the indicated biological functions of this attribute. Attributes that cannot directly link to biological functions were excluded in this analysis. Similar attributes were grouped together. '|' means either attribute was selected. For each attribute, genes that have customized classifiers with this attribute selected were enriched in GO terms that are related to the attribute, whereas genes that have customized classifiers without this attribute selected were not enriched in these GO terms. All the p values were adjusted by FDR method.

Attribute	GO term	p value	
		Attribute selected genes	Attribute non-selected genes
Conservation (GERP_RS | Phylop)	GO:0016071 mRNA metabolic process	4.3E-7	1
	GO:0044248 cellular catabolic process	6.0E-7	1
	GO:0006793 phosphorus metabolic process	1.4E-6	1
	GO:0016567 protein ubiquitination	8.4E-6	1
	GO:0008380 RNA splicing	1.3E-5	1
Active Site / Binding site (site)	GO:0005515 protein binding	<1E-30	1
	GO:0016874 ligase activity	<1E-30	1
	GO:0005102 receptor binding	<1E-30	1
	GO:0016567 protein ubiquitination	<1E-30	1
	GO:0032446 protein modification by small protein conjugation	<1E-30	1
Intramembrane (Intramem)	GO:0016021 integral component of membrane	<1E-30	1
	GO:0031224 intrinsic component of membrane	<1E-31	1
	GO:0044425 membrane part	<1E-32	1
	GO:0016020 membrane	<1E-33	1
	GO:0005886 plasma membrane	3.5E-21	1

Propeptide (propep)	GO:0034364 high-density lipoprotein particle	3.6E-14	1
	GO:0034361 very-low-density lipoprotein particle	1.9E-13	1
	GO:0034385 triglyceride-rich lipoprotein particle	1.9E-13	1
	GO:0032994 protein-lipid complex	1.2E-12	1
	GO:0034358 plasma lipoprotein particle	1.2E-12	1

Transmembrane (transmem)	GO:0042625 ATPase activity, coupled to transmembrane movement of ions	<1E-30	1
	GO:0019829 cation-transporting ATPase activity	<1E-30	1
	GO:0005261 cation channel activity	<1E-30	1
	GO:0008324 cation transmembrane transporter activity	<1E-30	1
	GO:0015399 primary active Transmembrane transporter activity	<1E-30	1

Polarity (polarity)	GO:0016874 ligase activity	<1E-30	1
	GO:0005515 protein binding	<1E-30	1
	GO:0003723 RNA binding	<1E-30	1
	GO:0008270 zinc ion binding	1.9E-26	1
	GO:0001664 G-protein coupled receptor binding	1.9E-24	1

Transmembrane tendency (TransmemTend)	GO:0022857 transmembrane transporter activity	<1E-30	1
	GO:0005215 transporter activity	<1E-30	1
	GO:0022891 substrate-specific transmembrane transporter activity	<1E-30	1
	GO:0015075 ion transmembrane transporter activity	<1E-30	1
	GO:0015293 symporter activity	<1E-30	1

	GO:0006397 mRNA processing	<1E-30	1

Flexibility (flexibility)	GO:0098609 cell-cell adhesion	1.0E-22	1
	GO:0016071 mRNA metabolic process	1.0E-22	1
	GO:0002376 immune system process	3.7E-14	1
	GO:0043484 regulation of RNA splicing	2.9E-13	1
Helix tendency (HelixTend)	GO:0044459 plasma membrane part	<1E-30	1
	GO:0005887 integral component of plasma membrane	3.3E-22	1
	GO:0005924 cell-substrate adherens junction	1.4E-17	1
	GO:0005912 adherens junction	1.9E-17	1
	GO:0005925 focal adhesion	4.5E-17	1
Coiled | CoilTend	GO:0043235 receptor complex	2.1E-11	1
	GO:0005856 cytoskeleton	5.9E-6	1
	GO:0030054 cell junction	1.9E-5	1
	GO:0044430 cytoskeletal part	2.1E-5	1
	GO:0097060 synaptic membrane	2.6E-5	1
Hydropathicity | Hydrophobicity	GO:0003723 RNA binding	<1E-30	1
	GO:0004872 receptor activity	5.8E-25	1
	GO:0005515 protein binding	5.8E-25	1
	GO:0005102 receptor binding	5.8E-25	1
	GO:0016874 ligase activity	9.6E-24	1
